# Enhanced Intracellular Photosensitizer Uptake and Retention by Targeting Viral Oncoproteins in Human Papillomavirus Infected Cancer Cells and Cancer Stem Cells

**DOI:** 10.3390/molecules28020647

**Published:** 2023-01-08

**Authors:** Elvin Peter Chizenga, Heidi Abrahamse

**Affiliations:** Laser Research Centre, Faculty of Health Sciences, University of Johannesburg, Johannesburg 2028, South Africa

**Keywords:** cancer stem cells (CSCs), human papillomavirus (HPV), side population (SP), non-side population (nSP), E6 oncoproteins, photodynamic therapy (PDT), SiHa cells, transformed, immunogenic

## Abstract

Immunogenic proteins in cancer are relevant targets for drug delivery. In Photodynamic Therapy (PDT), surface antigens have previously been used to deliver the photosensitizer (PS) to the tumor microenvironment for specific targeting. However, can we target intracellular antigens to achieve more than surface recognition? Can we possibly increase PS intracellular localization and prevent drug efflux at the same time? In this study, these questions were addressed by using a compound that can not only specifically recognize and bind to intracellular E6 oncoproteins in Human Papillomavirus (HPV)-Transformed cancer cells, but is also capable of enhancing transmembrane uptake using the cells’ own active transport mechanisms. HPV-transformed SiHa cells were cultured in vitro, and the resistant subpopulation was isolated using Magnetic Activated Cell Sorting (MACS). PDT was performed on four different cell types with varying physiognomies in terms of HPV oncoprotein expression and physiological form. Results demonstrated that tagging PSs on a carrier molecule that specifically delivers the PS inside the cells that express the target proteins enhanced both cellular uptake and retention of the PS even in the presence of drug efflux proteins on resistant subpopulations. These findings provide insight into the possibility of preventing cell-mediated resistance to PDT.

## 1. Introduction

Human Papillomavirus (HPV) causes about six different cancers in different tissues and organs of the body including the cervix uteri, vagina, vulva, penis, anus, and oropharynx, and, though less commonly, it is also associated with the pathologies of fourteen other cancer types including lung, breast, skin, eye and conjunctiva, colorectal, prostate, urinary bladder, urethra, etc., as a primary, or secondary agent in respective cases [1]. When HPV integrates itself with the host cells’ deoxyribonucleic acid (DNA), the resulting cellular transformation produces a phenotype of immortalized cells that express two main oncoproteins in their cytoplasm, the E6 and E7 oncoproteins, which also pass on to replicated generations [1,2]. These oncoproteins are immunogenic, hence provide an opportunity for the design of antibody (Ab)-mediated therapies or delivery systems that specifically recognize the E6/E7 expressing cells.

In the present day, Photodynamic Therapy (PDT) is being studied extensively for treatment of many localized cancers as an alternative for conventional chemo and radiation therapies. In fact, PDT is a good candidate for precancerous lesions and carcinoma in situ, e.g., the different stages of cervical intraepithelial neoplasia (CIN), to replace invasive therapies [3,4]. However, although PDT has proven more efficacious than conventional chemo and radiation therapies, it has not escaped the shrewdness of cancer cells with their ability to resist eradication. This inherent resistance to therapy, is mainly attributed to tumor heterogeneity which affects both the behavior of the tumors and their response to therapy. Resistant subpopulations of cells in the tumor possess characteristics that enable the cells to either avoid therapy by effluxing drugs before their action or by altering cell signaling after the drugs’ action [5]. In PDT, the efflux of PS and the alteration of cell signaling results in the inability of PDT to affect the cells, leading to failed treatment. Though most forms of PDT are under investigation in clinical trials and ex vivo experimentations, rising concerns of PDT resistance are being reported and addressing this issue early would hasten PDT approval and wide acceptability as an alternative to more lethal and invasive therapies. 

The E6 and E7 oncoproteins are exclusively present in the clone of cells replicating from the HPV-transformed mother cell [6]. Moreover, these proteins are very immunogenic and hence targetable using the corresponding anti E6/E7 monoclonal antibodies (mAbs) or their mimics. With the emergence of nanotechnology, the wide use of nanomaterials as carrier molecules to control the delivery of drugs and their action has changed the entire episteme of pharmacology, including PDT. Therefore, the incorporation of traditional PSs to carrier molecules alongside specific mAbs has the potential to enhance PDT in a way that it is possible to override cell-mediated resistance to therapy. In this present investigation therefore, a technique to enhance the efficacy of PDT in HPV-transformed cancers is reported. A multicomponent photo-active compound comprising of a phthalocyanine PS and anti E6 mAbs bound to a gold nanoparticle (AuNP) core was developed as previously described (Chizenga and Abrahamse, 2022) [7]. Here, this compound was used in vitro to enhance PDT by targeting intracellular anti-E6 oncoproteins in HPV-transformed cells and their resistant subpopulation known for drug/PS efflux due to the presence of ATP binding cassette (ABC) transporters on their cell membranes. This was undertaken to demonstrate the possibility of enhancing intracellular localization and retention of the PS in the presence of ABC transporter activity.

## 2. Results

### 2.1. Side Population Percentage Yield and Viability

The estimated viability of the isolated SiHa SP averaging at 80% is shown in Supplementary Material Table S1. Average percentage yield of isolated SP was approximately 0.93% of the total population.

### 2.2. ABC Transporter Activity and Stem Cell Marker 

**Figure 1:**
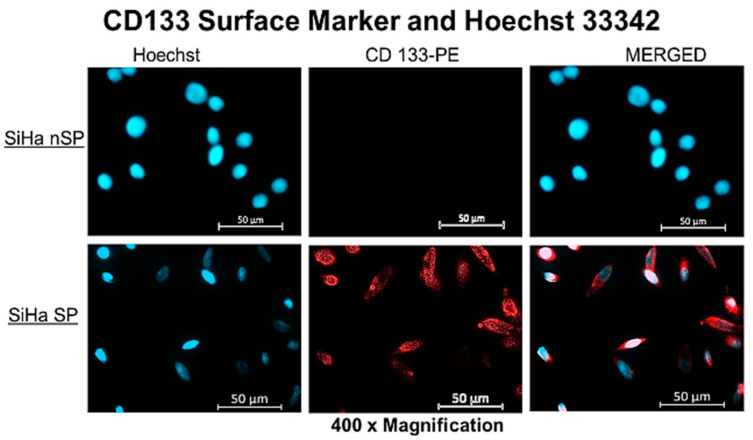
Fluorescent microscopy of the SP and nSP after treatment with Hoechst dye 33342, indicating low fluorescent signal in the SP and a high fluorescent signal in nSP.

Hoechst efflux and CD133 surface marker expression contrasted between the SP and the nSP. Figure 1 below presents the fluorescence signals captured for both nuclear Hoechst stain in light blue fluorescence and membrane CD133 PE in red. SP showed a lower fluorescence signal compared with the nSP. The fluorescence intensity is significant of the presence of the dye in higher concentrations. Hence the low intensity infers low dye concentration which is due to the efflux of the dye by the SP, owing to the presence of ABC transporters on the cell membrane. The semi-quantitative analysis of the detected signal to clarify the difference in intensity is included in Figure S2 to facilitate interpretation of Figure 1 below. Note that both cells having been treated with the same concentration of dye; the difference in intensity, i.e., low fluorescence signal in SP and high signal in nSP indicates the action of ABC transporters that pumped out the PS in the SP. The further staining of surface markers indicated the presence of CD133 antigens on the SP as detected by fluorescence signals, also shown in the figure below.

### 2.3. Enumeration of CD133 Expression of Cultured SP

**Figure 2:**
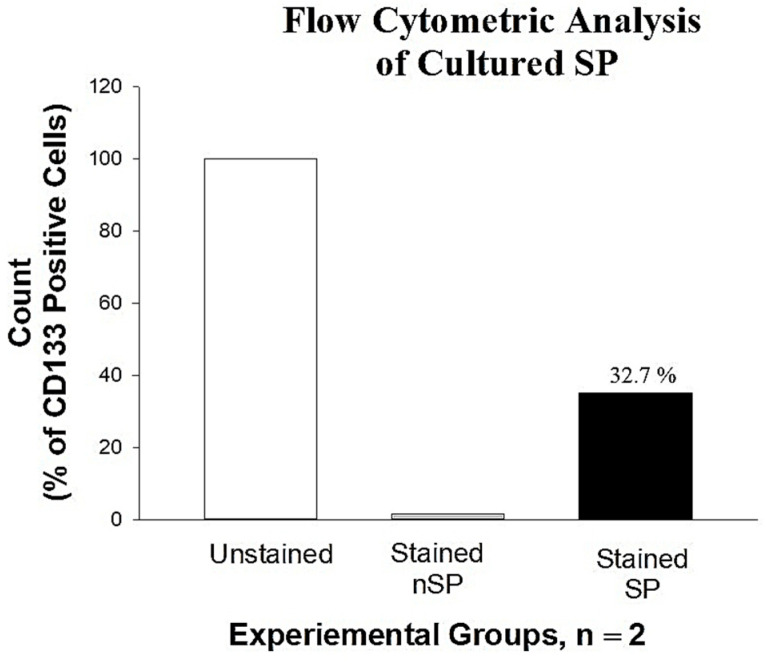
FCM analysis of the SP showing the expression of CD133 surface marker, indicating 32.7% positive for CD133. Chart was plotted using SigmaPlot software version 12. Note the decrease in CD133 expression due to proliferation of cells in culture.

Cells expressing the CD133 antigens were validated and quantified by FCM, as shown in Figure 2 below. Capturing 100,000 events per run, events showed 32.7% of PE fluorescence, indicating the presence of CD133 surface marker on the cells. The red fluorescence of cells stained with PE-anti CD133 was detected and analyzed using SigmaPlot version 12 (n = 2). Note the decrease in percentage of CD133 surface marker in cultured SP population after 7 days. This indicated proliferation of SCs to mature SiHa cells and consequent loss of stemness and SC antigens. Hence, all PDT experiments were performed using fresh isolated cells seeded directly on glass coverslips at low cell populations. 

### 2.4. Cell Viability, Morphology, and Stability in Co-Culture

**Figure 3:**
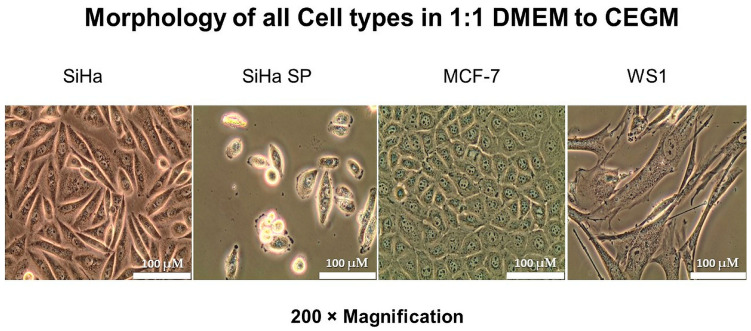
Cellular morphology of cells in optimum medium (1:1 DMEM to CEGM) indicating the morphological features of cells after adjusting medium formulation.

When cells were co-cultured in the 1.1 DMEM to CEGM media mix, their normal cell morphology was retained and viability was unaltered. Figure 3 below shows the morphology of the four cell lines in co-culture after 72 h of propagation. Cell viability was compared to the controls (i.e., the cells in their original recommended medium) and the variation in viability of >10% was allowed. Table 1 shows the percentage viability of the cells.

### 2.5. Cellular Uptake and Localization

**Figure 4:**
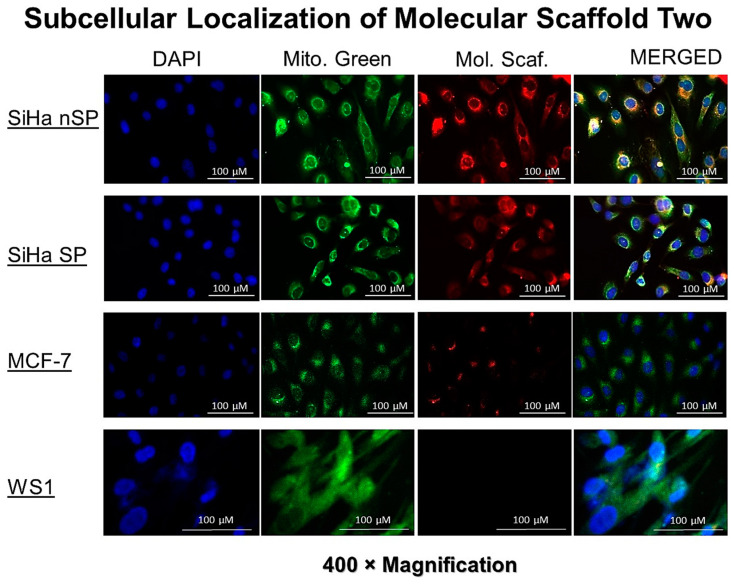
The fluorescence detection of the PS signal in the SiHa, SiHa SP, MCF7, and WS1 Fibroblast, indicating the uptake and localization of the molecular scaffolds in the cells. Different intensity can be seen and the intensity signals were detected as shown in the right column.

The uptake and localization showed the red auto fluorescence of the AlPcS_4_Cl. After co-culturing and treating the cells in a common environment, the difference in red auto-fluorescence can be clearly seen in the cells. Though all the cell populations except WS1 showed some red signal, the intensities were different. High intensity of the red fluorescence was seen in the SiHa nSP, followed by the SiHa SP, and very low red fluorescence was seen in the MCF7 cells. WS1 had no visible and/or measurable red fluorescence, as shown in Figure 4 below.

### 2.6. Post-Treatment Morphologic Assessment

**Figure 5:**
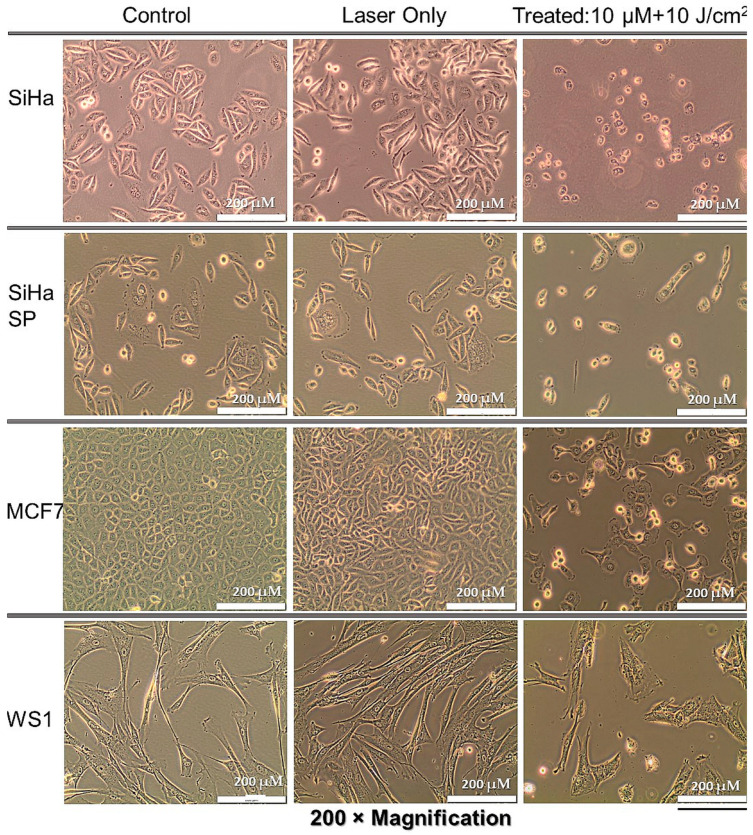
Post-treatment cellular morphology by light microscopy indicating the alteration in treated cells when compared to control cells and the laser only group.

Cells co-cultured the same way as experimental groups but not treated showed no changes in morphology, as seen in the first column of Figure 5 below. Similarly, the laser only group did not show any alteration in morphology, only slightly increased cell density due to the photobiomudulatory effect of irradiation in cells that did not receive the PS. However, the PDT-treated cells showed noticeable changes, with cell rounding, shrinking, and blebbing in the SiHa, SiHa SP, and MCF cells, with increasing damage, respectively. Because the concentration used was very low, not many cells died at the end of the 24 h, but all features pointed to terminal cell demise. The WS1 cells did not show substantial alterations in morphology at this concentration, possibly as complemented by the uptake studies where PS was not detected in the cytoplasm. Figure 5 below shows the observed findings.

### 2.7. Post-Treatment MTT Assay

**Figure 6:**
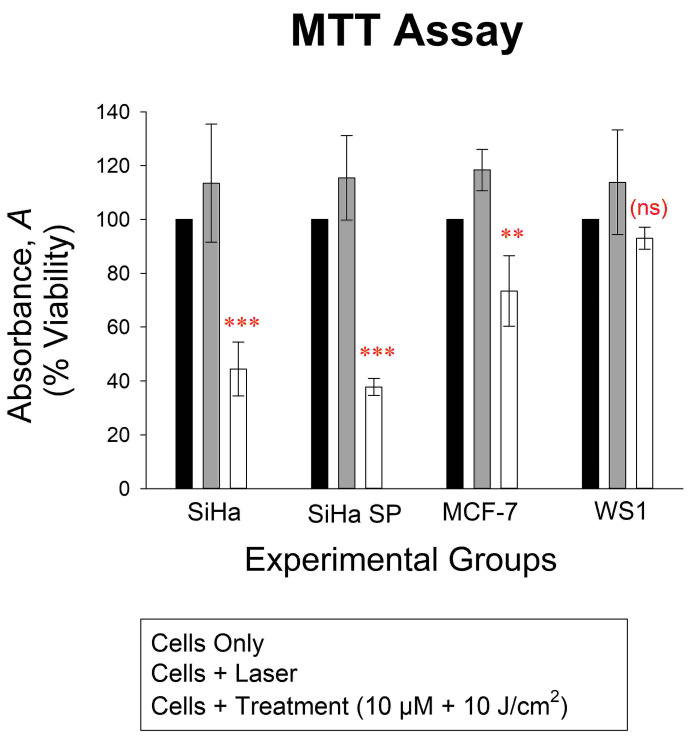
The viability of cells 18 h post-treatment showing significant decrease in viability of the SiHa and SiHa SP treated cells (*p* < 0.001, shown in the graph as ***), MCF-7 (*p* < 0.01, shown in graph as **), and no significant decrease (ns) in the WS1 cells, all cells as compared to the cells only control, which represented the 100% viability of cells.

Finally, in Figure 6 the MTT assay demonstrated the effect of PDT by induction of cytotoxicity and decreasing cell proliferation. The treatment of cells with the compound followed by irradiation showed increased cytotoxicity on the SiHa, SiHa SP, and MCF-7, with some alteration in WS1 cells, but it was statistically insignificant. The SiHa SP suffered the most damage (*p* < 0.001) according to the MTT assay, followed by the SiHA nSP and MCF-7 cells (*p* < 0.01).

## 3. Discussion

The need for new cancer therapies is prompted by the observed inefficiency of conventional therapies. The design of the new alternative therapies, therefore, should critically address the limitations of conventional therapies in order to fill up that gap. One of the causes of treatment resistance and cancer recurrence is the failure of conventional therapies to efficiently eradicate the stem cell (SC) population of the tumor, i.e., the cancer stem cells (CSCs). The mechanisms by which these CSCs resist therapy involve two main processes: effluxing drugs or alteration of cell signaling. Similarly, though widely accepted as a good alternative to invasive, lethal, and costly therapies, PDT has faced the same limitation where certain cancer cells have been reported to be resistant by both these mechanisms [5]. Opportunely, nanomedicine has since provided some solutions to this problem. When PSs are coupled to nanomaterials to alter the pharmacokinetics (PK) of the PS, PDT efficacy is substantially increased [8]. Moreover, incorporating mAbs onto the delivery vehicle alters both delivery of the PS and the pharmacodynamics at or in the tumors by virtue of selective binding to the specific antigens at the target site. This present study, therefore, investigated the effect of a newly designed compound comprised of the PS, anti-E6 mAbs, and PEGylated AuNPs to facilitate both PK and PD by specific delivery of the PS to HPV E6-expressing cancer cells, and facilitating uptake and retention.

AuNPs, due to their physicochemical characteristics, are able to deliver an increased PS payload to cells and facilitate uptake [9]. The novel compound here demonstrated the specific delivery of the PS to the target cells having simulated an in vivo microenvironment in vitro using a modified co-culture technique. In the presence of different cell types, those expressing the E6 oncoproteins showed increased uptake of PS and enhanced retention compared to those that do not express the proteins. Using very low concentrations, the normal cells represented by WS1 fibroblasts did not take up the PS as observed using fluorescence microscopy, did not undergo cytotoxicity after irradiation, and did not show any significant changes in cell morphology nor alteration in proliferation, indicating that this compound at the specified concentration did not have any effect on normal cells. Should this occur in vivo, it will imply that PDT using this compound may not damage any normal cells, thereby limiting side effects caused by unspecific reactions. Though the breast cancer MCF-7 cells showed some significant damage, their lack of E6 expression resulted in milder damage when compared to the other cancer cells that expressed the oncoprotein. MCF-7 cells probably took up some minute PS because of their state as highly porous cancer cells due to their increased LDL receptors on the membranes. However, because the compound was functionalized with mAbs not relevant for the cells, concentrations absorbed were negligible. 

In the HPV E6-expressing SiHa cells, however, at a very low concentration, sufficient PS was detected in the cytoplasm of the cells. SiHa CSCs were previously confirmed to possess ABC transporters that pump drugs, dyes, and PSs out of the cytoplasm by their transporter activity. Here, it was demonstrated for the first time that CSCs absorbed sufficient PS similar in proportion to that absorbed by the nSP which do not have efflux ability. Using nude PS, i.e., the PS alone without a delivery vehicle, the PS would be effluxed by the cells, as reported previously by our team [10] and others [11,12]. However, because this time the PS was bound to a carrier molecule with specific binding to intracellular proteins, intracellular time was increased by preventing efflux. In the end, the SP also showed increased damage after irradiation, further proving the retention effect of the compound with the subsequent induction of terminal cell death after irradiation. 

Based on these findings, therefore, this compound is confirmed to be one that has a multifactorial purpose, facilitating both PK and PD. What is important to note in these observations is that at the structural level of cells, the movement of the compound in the intracellular compartment is vital for understanding efflux or retention. The notion about cells is that the cytoplasm is a fluid compartment. However, somatic cells are complex, with a dense membranous intracellular compartment composed of microtubules and filaments that influence the transcytosis of compounds within the cells. When compounds enter the cells, they still need to move around within by transcytosis to reach the target organelles and/or the nucleus. With PS efflux, the possible causes of immediate efflux would be the trapping of absorbed PS within the interior perimembrane region, making it easy for ABD transporters and P-glycoproteins to affect the efflux of the PS. This is what happens when nude PSs are used. In HPV-transformed cells, E6 oncoprotein is mainly located in the nucleus, perinuclear region [13], and co-localizes along with the apoptosis-inducing factor (AIF) due to its inhibitory role on the caspase-dependent pathway [14]. Additionally, some AIF is primarily localized in the cytosolic side of the outer mitochondrial membrane with the majority in the intermembrane space of the mitochondria. Fluorescence imaging indicated intracellular accumulation of the compounds, which are seen to accumulate in the regions where the E6 oncoproteins localize in the cells. As theorized, the compounds moved by transcytosis and were able to bind with the E6 proteins via the immunological interactions between the E6 mAbs and the antigenic sites of the oncoproteins. Hence, this evidence shows that the interaction of the mAb with the oncoproteins aided in the prevention of potential efflux, resulting in PS accumulation inside the cells and subsequent cytotoxicity of both the SP and nSP of the SiHa cell line. 

## 4. Materials and Methods

### 4.1. Compound Synthesis

The compound used in this investigation was designed, synthesized, and characterized as reported in Chizenga and Abrahamse, 2022 [7], and summarized in Supplementary Material Figure S1. The compound was comprised of Aluminum (III) Phthalocyanine Chloride Tetrasulfonic Acid, AlPcS4Cl, molecular weight 895.2 g/mol (Frontier Scientific Inc. Logan, UT, USA) and Anti HPV16 + HPV18 E6 [C1P5] ab70 (Abcam, Cambridge, United Kingdom) with a size of 16.5 kDa loaded onto carboxylic acid factionalized, PEG 3000 coated gold nanoparticles of 10 nm in size (PEGy-AuNPs) (Product No. 765430), OD50 of unspecified Molar concentration. The new three-part compound that was developed contained a PS concentration of 457.7 µM and was used as such.

### 4.2. Cell Culture and Side Population Separation 

For SiHa cells, the American Type Culture Collection, ATCC^®^ HTB35™ cell, an E6+ HPV-transformed cell line (ATCC, Manassas, VA, USA), was cultured in Dulbeco’s Medium Essential Medium, DMEM (Sigma-Aldrich, Johannesburg, South Africa) at 37 °C in a humidified incubator, with 5% CO_2_ atmosphere and 85% humidity. Following successive subculturing to increase cell population, the SiHa side population (SP), i.e., the cancer stem cells (CSCs), was separated from the total population by magnetic-activated cell sorting (MACS) using the CD133 MicroBead Kit, human-lyophilized (Lot#. 5220401024). Separation of the SP was performed at 4 °C. The cells were centrifuged at 300× *g* for 10 min and then re-suspended in 300 mL of isolation buffer at pH 7.4 to a final concentration 1 × 10^8^ cells in 2 mL Eppendorf tubes. To each tube containing 300 µL isolation buffer per 1 × 10^8^ cells, 100 µL of FcR blocking reagent was added and mixed by brief vortexing. Subsequently, 100 µL of the anti-CD133 conjugated magnetic microbeads was added to the cell suspension in blocking reagent and incubated at 4 °C with continuous rotation using the MACSmix tube rotator (Miltenyi Biotec, Bergisch Gladbach, Germany). After tagging, the cells were washed once with isolation buffer by adding 1.5 mL of buffer and centrifuging at 300× *g* for 10 min. The supernatant was removed and the cells were re-suspended in 0.5 mL of buffer. Magnetic separation was performed using LS columns on a QaudroMACS^TM^ Separator. The separated SP and the flow-through non-side population (nSP) were both re-cultured in DMEM and Cervical Epithelial Growth Medium (CEGM) (Cat. # C0013-29, Separation Scientific SA (Pty) Ltd., Roodepoort, South Africa), respectively. Estimation of the percentage yield of the SP separated from the nSP and viability were performed using the automated cell counter Countess^®^ II FL (Invitrogen, Waltham, MA, USA) after staining with trypan blue. 

### 4.3. Characterization of the SP and nSP

#### 4.3.1. ABC Transporter Activity and Surface Marker Validation 

The characterization of the SP and nSP to determine their properties and confirm their biological identity was performed using fluorescence microscopy for qualitative detection of the CD133 surface marker and membrane dye efflux potential of the SP expressing ABC transporters. Efflux potential and surface marker validation were assessed simultaneously by staining cells cultured at a concentration of 2 × 10^5^ per 22 × 22 mm coverslips with 3 µg/mL of Hoechst 33342, trihydrochloride, trihydrate, (MW 615.99) (H3570) and 25 µL of CD133/1 antibody, anti-human, PE (Meltinyi Biotec, North Rhine-Westphalia, Germany, REAfinity™, LOT# 5220401828). To achieve this, the seeded cells were first treated with Hoechst stain at physiological conditions for 45 min, after which Hanks Balances Salt Solution (HBSS) (Sigma Aldrich, Johannesburg, South Africa) was used to wash. The cells were re-incubation for another 30 min after washing to allow for the cells’ physiological effect on the Hoechst stain. The cells were then washed once and then fixed with cold 4% paraformaldehyde. Staining with CD133/1 antibody in 200 µL blocking buffer was performed for 45 min at 4 °C in the dark. The stained cells were washed three times with wash buffer (0.1% Triton X in PBS). Images of the cells were captured using the Carl Zeiss Axio Z1 fluorescence microscope with 359 Ex/461 Em filters for Hoechst and 649 Ex/670 Em for PE. 

#### 4.3.2. Quantification of CD133 Cells Using Flow Cytometry

Quantitative estimation of cells expressing the CD133 to confirm stemness of the SP was performed using flow cytometry (FCM). Samples were prepared by prior fixation using 200 µL of 4% paraformaldehyde for 10 min at room temperature. The fixed cells were washed two times with cold PBS by adding 1 mL of PBS each time followed by centrifugation at 500× *g* for 5 min. The sediment was reconstituted in 100 µL of PBS, and 100 µL of blocking solution was added to prevent nonspecific binding of the antibodies. The cells were stained with 20 µL of PE-conjugated anti-CD133 in 200 µL of PBS and incubated in the dark for 30 min with continuous rotation using the MACS mix tube rotator placed in the refrigerator. After staining, the cells were washed three times with cold PBS. The stained cells were then resuspended in 300 µL of PBS for FCM analysis using the BD Accuri C6 Flow Cytometer (BD Biosciences, Johannesburg, South Africa).

### 4.4. Growth Medium Optimization and Co-Culture

To clearly establish the role of anti E6 mAbs, four cell types with varying physiognomies in relation to E6 expression and physiological status, were co-cultured in a modified chamber supplied with culture medium comprised of a 1:1 mixture of DMEM and CEGM supplemented with 5% FBS. Besides the SiHa SP and nSP described above, WS1 cells, (ATCC^®^ CRL-1502TM, Manassas, VA, USA) a fibroblast cell line to represent normal noncancerous cellsE6 that are negative for the E6 oncoprotein, and MCF-7 (ATCC^®^ HTB22™), a breast cancer cell line to represent a cancerous but E6 negative cell type, were included (summarized in Table 2 below).

Before co-culturing, the cells had to be adapted to and standardized for a common medium. All these cell lines are anchorage dependent and were cultured as monolayer cultures. To achieve this optimization, cells from each culture were first grown separately in their usual medium, followed by a systematic change in formulation, progressively, as recommended by the ATCC [15]. SiHa and MCF7 grow well in DMEM with 10% FBS; WS1 grew well in MEM with 10% FBS; and SiHa SP grew well in CEGM. The formulations of medium were changed progressively, starting with 1:3 DMEM in CEGM, while observing morphology moving down to 1:2 and 1:1 split ratios for SiHa, MCF-F, and WS1 cells. The isolated SiHa SP were adapted using similar split ratios, however, starting from CEGM to DMEM. Ultimately, the most conducive medium mix for all four cells was adapted and proceeding experiments were conducted in it. To proceed to co-culture, each cell type was first grown separately at a concentration of 2 × 10^5^ cells on 22 × 22 glass coverslips, placed in 8.8 cm^2^ tissue culture plates, and supplied with culture medium mix for 12 h to ensure complete attachment to the coverslips. After attachment, the coverslips were washed once with 1 mL warm HBSS. A sterilized 56.7 cm^2^ petri dish was labelled and compartmentalized with sterile applicator sticks. Each coverslip was then placed in each designated compartment using sterilized forceps and 10 mL of media was added to have all cells completely submerged into the media. 

### 4.5. Intracellular Uptake, Localization, and Retention 

Florescence microscopy was used to detect the uptake and localization of the compound. Co-cultured cells as described were treated with 10 µM of the compound. The cells were incubated for 12 h, after which they were washed three time with warm HBSS. Re-incubation for another hour after washing was performed by adding fresh pre-warmed culture media to the incubator to allow for further removal of unabsorbed P, and possible PS efflux by the SiHa SP. The cells were then washed twice with HBSS followed by fixation with cold 4% paraformaldehyde. The cells were then stained with 100 nM mitotracker (Invitrogen M7514) and counterstained with 4′6-diamidine-2-phenylindole DAPI solution at a concentration of 2 µg/mL. Images were captured using 40× oil immersion objective on a Carl Zeiss Axio Z1 microscope (Carl Zeiss MicroImaging GmbH, Aalen, Germany), with 490 Ex/516 Em filters for green and 649 Ex/670 Em for red fluorescence. 

### 4.6. Photodynamic Treatment of Co-Cultured Cells 

Co-cultured cells were first treated with 10 µM concentrations of the compound. After 12 h, the coverslips cells were removed from the co-culture vessel to 8.8 cm^2^ culture plates to proceed with each separately. Note that the co-culture was utterly important for uptake of the compound only, to establish selectivity of the compound, and proceeding experiments including irradiation and assessment were conducted in individual coverslips. The cells were washed three times with HBSS, followed by re-submersion in 1 mL of fresh media in 8.8 cm^2^ culture plates. Irradiation was performed using a semiconductor diode laser device, emitting a continuous wave, with a wavelength of 673.2 nm, a power output of 98.0 mW, and a spot size of 9 cm^2^ (Oriel Corporation, National Laser Center (NLC), Johannesburg, South Africa). Irradiation was performed at the fluence of 10 J/cm^2^ at room temperature. Post-irradiation, the cells were re-incubated in 3 mL of media 12 h for morphological assessment and 24 h for proliferation assay as described further below. 

#### 4.6.1. Morphologic Assessment

Assessment of possible alterations in cell structure and monolayer disruption was performed using the inverted light microscopy. Cells were observed at 12 h post-irradiation using an Olympus inverted light microscope (Wirsam Scientific, Johannesburg, South Africa, CKX41) with a built-in camera. 400× magnification was used to view the morphological appearance of the cells assessing the size, shape, and adherence to surface of the dish and images were captured and analyzed using ImageJ software 1.46 r version (Public Domain, BSD-2 Online, developed by the National Institute for Health, NIH, Bethesda, MD, USA) and Ms PowerPoint 2016 (Licensed for University of Johannesburg, Johannesburg, South Africa) for reporting.

#### 4.6.2. MTT Assay

The MTT [3-(4,5 4,5-dimethylthiazoldimethylthiazol-2-yl)-2,5-diphenyl tetrazolium bromide] assay was conducted to determine PDT toxicity. Treated cells as described were cultured for 24 h post-PDT. After the 24 h incubation, the wells were washed once with pre-warmed HBSS and the cover slips were removed and put in new culture plates. Then, 100 μL of cell detachment solution and the detached cells were directly pipetted into a clear bottom 96 well plate. To each well, 100 μL of 1× MTT (Elabscience Biotechnology, Texas, USA, Cat #E-CK-A341) working solution was added, and the plates were incubated at culture conditions for 4 h. After the 4 h, 150 μL DMSO was added to each well to dissolve the formazan. The plate was shaken using a flat shaker for 5 min. Absorbance was then determined using a UV–Vis spectrophotometer at 570 nm.

### 4.7. Statistical Analysis 

All experiments were repeated three times (n = 3). Quantitative assays were performed in duplicate, and an average of the results was used, while repeats of qualitative experiments including images were randomly selected for presentation. Statistical analysis was performed using SigmaPlot software version 12.0 (Systat Software, Inc., Chicago, IL, USA) for MTT assay and MS 2016 was used for calculation of cell yields and viability. The mean, standard deviation, standard error, and significant changes were calculated, with the student t-test performed to determine the statistical difference between the control and experimental groups. Statistically significant difference is shown in graphs as (*) for *p* < 0.05, (**) for *p* < 0.01, and (***) for *p* < 0.001. The standard error on all plotted graphs is represented by error bars.

## 5. Conclusions

In summary, it is evident that there is a genuine need for therapies that are efficacious enough to mitigate the observed resistance when treating cancer. With the wide use of nanomaterials and the presence of mAbs that can be incorporated for delivery of PSs to the cells, alongside facilitating uptake and retention, it is possible to deliver PS load that supersedes the inherent resistance pattern by the cells. This investigation reported on a methodology for enhancing intracellular uptake and retention in HPV-transformed cells by using specific mAbs to the E6 viral oncoproteins. With the presence of other types of immunogenic proteins in other cancer types, such approaches still can be modified per application and enhance therapeutic effect in every given situation. Thus, future studies should seek to investigate immunogenic protein in cells, to use them as targets for delivery of PSs for PDT and/or other drugs. 

## Figures and Tables

**Table 1:** Viability of cells in 1:1 medium formulation compared against original medium, respectively.

Cell Line	Viability in Original Medium	Viability in 1:1 (DMEM to CEGM) Medium	Variation
SiHa	92%	85%	−7
SiHa SP	82%	84%	+2
MCF-7	94%	90%	−4
WS1	78%	82%	+4

**Table 2:** Experimental (SiHa Sp and nSP) and Control (WS1 and MCF-F) groups used.

Cell Line	Features	Purpose
SiHa nSP	E6+ HPV transformed cell line	HPV E6 Experimental
SiHa SP	Resistant E6+ HPV transformed cell line, ABC transporter +	HPV E6 Experimental
MCF 7	Cancerous, HPV E6 –	HPV E6 Control group
WS1	Normal noncancerous, HPV E6 –	HPV E6 Control group

## Data Availability

Data supporting the findings of this study are stored in the University of Johannesburg Library Data Repository. Raw data and/or link to repository are available from the corresponding author on request.

## Supplementary Materials

The following supporting information can be downloaded at: https://www.mdpi.com/article/10.3390/molecules28020647/s1, Figure S1: Summary of Conjugation Method and Characterization; Figure S2: Quantitative fluorescence intensity for interpretation of Figure 1. Table S1: Percentage Yield Calculation.

## Sample Availability

Samples of the compounds are not available from the authors.
