# Supplementary material for: Enhanced Intracellular Photosensitizer Uptake and Retention by Targeting Viral Oncoproteins in Human Papillomavirus Infected Cancer Cells and Cancer Stem Cells
Source: Molecules. 2023 Jan 8;28(2):647. doi: 10.3390/molecules28020647 (PMC9867367; doi:10.3390/molecules28020647)
Supplement: Supplementary file 1 [file molecules-28-00647-s001.zip › molecules-2083987-supplementary.pdf]

Enhanced Intracellular Photosensitizer Uptake and Retention by Targeting Viral Oncoproteins in Human Papillomavirus Infected Cancer Cells and Cancer Stem Cells

Elvin Peter Chizenga and Heidi Abrahamse *

Supplementary Materials

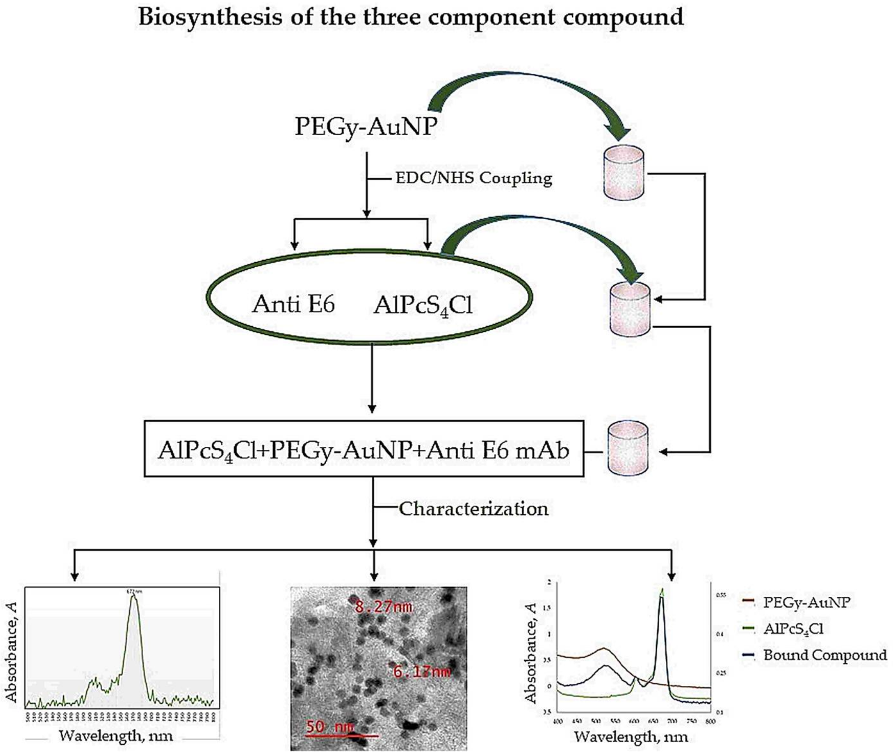

Figure S1. Summary of conjugation method.

$$\%Yield = \frac{Cell\ count\ after\ isolation}{Total\ cell\ count} \times 100$$

Table S1. Percentage Yield Calculation.

Run 1	Run 2	Run 3
$\%Yield = \frac{9.73 \times 10^5}{9.34 \times 10^8} \times 100$ $\%Yield = 1.04\%$	$\%Yield = \frac{8.09 \times 10^5}{8.37 \times 10^7} \times 100$ $\%Yield = 0.97\%$	$\%Yield = \frac{2.84 \times 10^5}{3.6 \times 10^7} \times 100$ $\%Yield = 0.79\%$

The average yield = $\frac{(Run\ 1+Run\ 2+Run3)}{3}$

= 0.93%

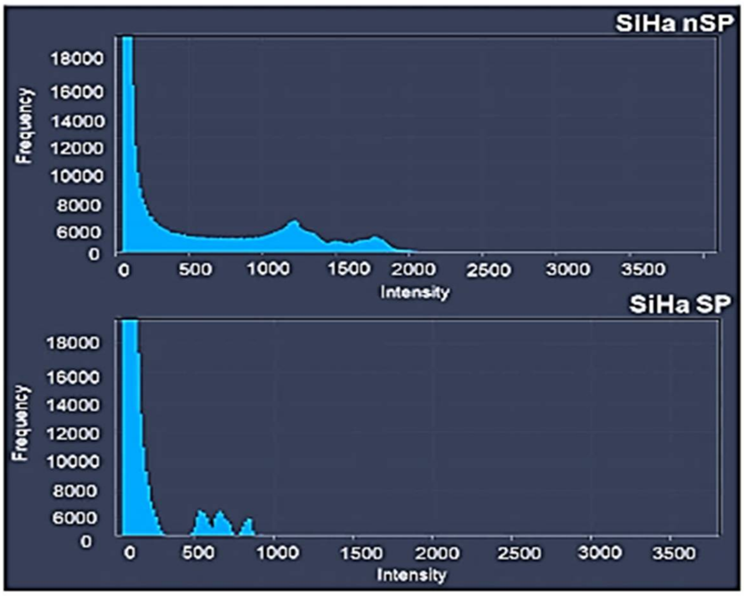

Figure S2. Percentage Yield Calculation for interpretation of Figure 1. Hoechst signal quantification in the nSP (top) and SP (bottom) as detected and enumerated by intensity detection on the Carl Zeiss microscope. Intensity is plotted on the x-axis, and the nSP shows more Hoechst intensity as opposed to the low intensity of the SP.
